# Equine Placentitis in Mares Induces the Secretion of Pro-Inflammatory Cytokine *e*IL-1β and the Active Extracellular Matrix Metalloproteinase (MMP)-9

**DOI:** 10.3390/vetsci10090532

**Published:** 2023-08-22

**Authors:** María Margarita Morales-Vázquez, Europa Meza-Serrano, Irlando Lara-Pereyra, Ricardo Josué Acuña-González, Rogelio Alonso-Morales, Sergio Hayen-Valles, Ana Myriam Boeta, Luis Zarco, Jair Lozano-Cuenca, Jorge Skiold López-Canales, Héctor Flores-Herrera

**Affiliations:** 1 Departamento de Immunobioquímica, Instituto Nacional de Perinatología “Isidro Espinosa de los Reyes” INPerIER, Ciudad de México 11000, CP, Mexico; margarita.morales.mvz@gmail.com (M.M.M.-V.); acugyomdik@gmail.com (R.J.A.-G.); 2 Departamento de Reproducción, Facultad de Medicina Veterinaria y Zootecnia, Universidad Nacional Autónoma de México, Ciudad Universitaria, Ciudad de México 04510, CP, Mexico; europosky@gmail.com (E.M.-S.); kekohayen@prodigy.net.mx (S.H.-V.); amyriam@unam.mx (A.M.B.); 3 Departamento de Ginecología, Hospital General de Zona 252, Instituto Mexicano del Seguro Social, Atlacomulco 28984, Mexico; 4 Genética, Laboratorio de Biotecnologías, Facultad de Medicina Veterinaria y Zootecnia, Universidad Nacional Autónoma de México, Ciudad Universitaria, Ciudad de México 04510, CP, Mexico; ralonsom@unam.mx; 5 Centro de Enseñanza, Investigación y Extensión en Producción Ovina, Facultad de Medicina Veterinaria y Zootecnia, Universidad Nacional Autónoma de México, Tres Marías, Ciudad de México 62515, Mexico; lazq@unam.mx; 6 Departamento de Fisiología y Desarrollo Celular, Instituto Nacional de Perinatología “Isidro Espinosa de los Reyes” INPerIER, Ciudad de México 11000, Mexico; prayax@hotmail.com (J.L.-C.); skiold666@hotmail.com (J.S.L.-C.); 7 Sección de Estudios de Posgrado e Investigación, Escuela Superior de Medicina del Instituto Politécnico Nacional, Ciudad de México 11340, Mexico

**Keywords:** equine placentitis, extracellular matrix metalloproteinases, foal health, pregnant mares, inflammatory cytokine

## Abstract

**Simple Summary:**

Equine placentitis is associated with abortions, mortality of mares, and foal deaths and produces economic losses for the equine industry. The diagnosis of equine placentitis often occurs long after the establishment of the disease, for which treatments are ineffective. Ultrasound is an excellent tool for detect fetal and placental changes; however, it is not accessible in all cases, and specialized training is required. In this study, we demonstrate that *e*IL-1β levels in the serum of mares with ultrasonographic signs of placentitis can be used as biomarkers of disease severity and its probable impact on the health and viability of the foal. We did not find significant differences in the activity of *pro*MMP-9 in the serum of mares with placentitis, so new studies will be required to validate *pro*MMP-9 as a biomarker.

**Abstract:**

Equine placentitis is characterized by infection and inflammation of the placenta. Different biomarkers associated with this inflammatory response have been evaluated in experimentally induced equine placentitis, but not in pregnant mares with spontaneous placentitis. The aim of the current study was to determine the concentration of *e*IL-1β and the activity of *pro*MMP-2 and *pro*MMP-9 in the serum of healthy mares and mares with placentitis on days 240 and 320 of gestation to explore whether these biomarkers are associated with equine maternal placentitis and/or with the birth of an infected or inviable foals. Serum samples were collected from sixteen pregnant English Thoroughbred mares, retrospectively classified as follows: (1) healthy mares with full-term gestation; and (2) mares with ultrasonographic signs of placentitis. The health of each foal was examined at birth, and it was decided to classify the cases into four groups: (1) healthy mares delivering a healthy foals (HM-HF, *n* = 6); (2) mares with USP delivering a healthy foal (USP-HF, *n* = 3); (3) mares with USP delivering a live septic foal (USP-LSeF, *n* = 4); and (4) mares with USP delivering a dead foal (USP-DF, *n* = 3). *e*IL-1β was quantified by ELISA, and *pro*MMP-2 and *pro*MMP-9 activity by gelatin zymography electrophoresis. In healthy mares, the serum concentrations of *e*IL-1β underwent a significant 16.5-fold increase from day 240 to day 320 of gestation. Although similar results were found in the mares with ultrasonographic signs of placentitis that delivered a healthy foal, those delivering a live septic or nonviable foal exhibited much higher concentrations of eIL-1β. *pro*MMP-2 and *pro*MMP-9 activity was not associated with maternal placentitis, foal infection, or death. Hence, the presence of placentitis severe enough to affect the health of the foal can be confirmed or discarded by determining the *e*IL-1β concentration in mares that have shown ultrasonographic signs of placentitis.

## 1. Introduction

Equine placentitis is characterized by inflammation of the placenta caused by infection with pathogenic microorganisms [1,2], which can reach the placenta by two pathways. Most commonly, pathogens ascend from the vagina into the uterine lumen before invading placental membranes [3,4,5,6]. Less frequently, pathogens or microbial products are transported from the oral cavity (or other point of entry) to the placenta through the bloodstream [7,8,9,10]. Equine placentitis is linked to premature myometrial activation [11], and consequently, with abortions [12,13,14]. This disease has been associated with the mortality of mares [15] and neonatal foals [16,17], among other conditions.

The causal agents of equine placentitis are Gram-positive (*Streptococcus equi* subsp. zooepidemicus, *Streptococcus equisimilis*, *Staphylocossus* spp., *Corynebacterium pseudotuberculosis*, and *Pseudonocardia* spp.), and Gram-negative bacteria (*Escherichia coli*, *Pseudomonas* spp., *Klebsiela* spp., *Salmonella abortus* equi, and *Chlamydia*) [2,18,19], virus (gammaherpesvirus and equine herpervirus) [17,20], and fungus (*Aspergillus terreus*) [21]. Toll-like receptors (TLRs) in placental cells recognize the molecular pattern associated with many of these pathogenic microorganisms [22]. Recently, Hossam El-Sheikh Ali et al. (2021) experimentally induced the development of placentitis in six pregnant Equus caballus mares with *S. equi* ssp. *zooepidemicus* and found a higher expression of TLR-2 and TLR-7 in the myometrium and placenta [23,24]. The interaction between TLRs and bacterial pathogens activates the MyD88/IRAK1/TRAF signaling pathway, which activates the nuclear transcription-regulated factor kappa B (NF-ĸB) [25,26]. Finally, NF-ĸB is a critical component of several cytokine signaling pathways [27,28]. Based on the experimental induction of equine placentitis, different biomarkers have been identified that are related to the inflammatory response (IL1β, IL-6, IL-8, and TNFα) [11,29] and to premature activation of myometrial contraction (PGE2, PGE2α, and oxytocin) [5,23,30,31].

Interleukin type 1-beta (IL-β) is a pivotal cytokine in several second messenger signaling pathways in both physiological and pathological processes [32]. IL-1β acts as a modulator during ovulation, in oocyte maturation, during early embryonic development [33,34], and in the activation of the inflammatory response [28,35,36]. It also acts as a modulator of specialized cells of the immune system [11,30] and induces the expression of matrix metalloproteinases (MMPs) [37,38].

In various physiological processes, the expression of MMPs has been associated with cell migration [39,40], angiogenesis [41,42,43], and implantation [44,45], while in several equine pathological conditions, it is linked to an inflammatory response [37,46,47]. Recently, Hossam El-Sheikh Ali et al. (2020), using an experimental model of equine placentitis, found an increase in the expressions of MMP-1 and MMP-8, which was associated with the expressions of TLR-2 and TLR-7 [23,24]. MMPs, are a family of zinc-dependent endopeptidases and produced in several physiological and pathological conditions by a wide variety of cell types, including neutrophils [48,49], macrophages [47], leukocytes [50], bronchial epithelial cells [46,51], and equine endometrial cells [23,52]. These endopeptidases induce the degradation of various structural components of the extracellular matrix, including collagen types I, IV, V, VII, and X, fibronectin, elastin, proteoglycan [53,54], the basement membrane [55], and cell-binding adhesion proteins [56,57].

The substrates degraded by MMPs provide the basis for their classification. Among commonly known are stromelysin-1 (MMP-3), -2 (MMP-10), and -3 (MMP-11); collagenase-1 (MMP-1), -2 (MMP-8), and -3 (MMP-13); gelatin-A (MMP-2) and -B (MMP-9); matrilysin type I (MMP-7) and type II (MMP-26); and membranal type I (MMP-14, -15, -16, and -24) and type II (MMP-23) [58,59,60]. The chromosomal location of all MMPs has been determined in all species with a reference-quality genome [61].

Although there are experimental models of placentitis in mares, the expressions of inflammatory and degradative biomarkers during equine physiological pregnancy and pregnancies with spontaneous placentitis is still unknown. It has been reported that inoculating pregnant mares with *Streptococcus equi* ssp. *zooepidemicus* activates the expression of prostaglandins, inflammatory cytokines, at 290 days of gestation [4]. Therefore, it would be important to know if the inflammatory response is activated prior to 290 days of gestation. Thus, the aim of the current contribution was to analyze the concentrations of *e*IL-1β and the activity of *pro*MMP-2 and *pro*MMP-9 in the serum of mares with and without placentitis at 240 and 320 days of gestation, and to determine if these biomarkers are related to placentitis and foal mortality.

## 2. Materials and Methods

### 2.1. Ethics Committee Approval

This protocol was approved by the Ethics Committee on Animal Experimentation at the Universidad Nacional Autónoma de México (SICUAE.MC-2020/2-5).

### 2.2. Animals and Experimental Design

**Figure 1:**
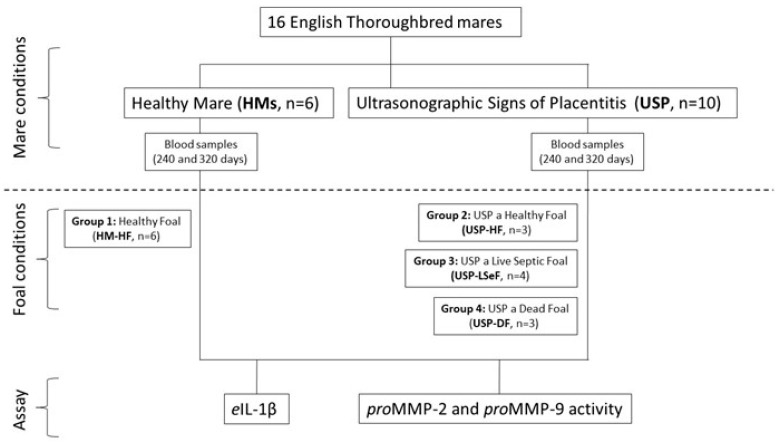
Schematic diagram of the study design. According to the conditions of the mares, they were classified as healthy mares (HM; *n* = 6), or mares with ultrasonographic signs of placentitis (USP; *n* = 10). In each case, the health of the neonatal foal was evaluated, and they were subclassified into four groups: HM-HF (*n* = 6), USP-HF (*n* = 3), USP-LSeF (*n* = 4), and USP-DF (*n* = 3). The quantification of *e*IL-1β and the activity of *pro*MMPs was evaluated in the serum from mares at 240 and 320 days of gestation.

Figure 1 shows the study design, the classification of mares and the subclassification based on foal health. Sixteen pregnant English Thoroughbred mares (11.7 ± 4.4 years old; weighing 500–600 kg) were included in the study, all underwent medical surveillance throughout the gestation period.

According to the medical surveillance and the condition of each placenta recovered after delivery, the mares were assigned to one of two groups: (1) healthy mares with full-term gestation (HMs, *n* = 6); and (2) mares with ultrasonographic signs of placentitis (USP, *n* = 10), indicated by a utero-placental thickness greater than 7 mm on day 240 of gestation and/or greater than 10 mm on day 320 [3,62]. In every case, the placentas were recovered immediately after delivery and evaluated for the presence of edema, congestion, necrosis or purulent, local, or generalized exudate [5,63].

In all cases, the health of the foal was also examined at birth to ascertain the presence or absence of sepsis [64]. The foals were classified as healthy foals (HF), or live septic foals (LSeF) if they fulfilled any or all of the following criteria: (1) those who, in the first 24 h of life, presented alterations in their normal behavior, such as discomfort when sleeping, stopping nursing, and diarrhea, or alterations in their general physical examination, taking as normal ranges a heart rate of 80–110 beats/min, respiratory distress with 25–30 breath/min, fever of >38 °C, urinary density of 1.010–1.015, pink mucous membranes, and capillary filling time of 1 s; (2) >1 site of placenta infection based on weight and external evaluation. Dead foals (DF) were those who presented the alterations described above during the first 24 h of birth, underwent treatment, and died; this group included a fetus that was aborted and a foal that was born by placenta previa, alive but weak, and at the general physical examination, its heart frequency was very low, so euthanasia had to be performed [65,66,67].

Considering the health of the mares and that of their foals, it was decided to classify the cases into four groups: (1) healthy mares delivering a healthy foal (HM-HF, *n* = 6); (2) mares with USP delivering a healthy foal (USP-HF, *n* = 3); (3) mares with USP delivering a live septic foal (USP-LSeF, *n* = 4); and (4) mares with USP delivering a dead foal (USP-DF, *n* = 3), defined by organ dysfunction, subcutaneous edema, and conjunctivitis [15] (Figure 1).

### 2.3. Blood Samples

Blood samples were obtained from 16 mares at 240 and 320 days of gestation by jugular vein puncture and collected in a Vacutainer tube (BD, Franklin Lakes, NJ, USA). The blood samples were immediately centrifuged at 1500 rpm for 10 min at room temperature. The serum was separated and stored at −70 °C until it was used to measure the concentration of *e*IL-1β and the activity of *pro*MMP-2 and *pro*MMP-9.

### 2.4. eIL-1β Assay

The serum levels of *e*IL-1β were quantified by a specific Douset ELISA assay (DY3340, R&D system, Minneapolis, MN, USA), as previously described [68]. A standard curve was constructed from 125 to 8000 pg/mL. The final concentration of the total protein per sample was expressed in pg.

### 2.5. Zymography Gel Activity

The activity of *pro*MMP-2 and *pro*MMP-9 was determined by zymography gel, as previously reported in other models of infection [68,69]. SDS-polyacrylamide gels (4%) were co-polymerized with porcine gelatin (1 mg/mL, substrate gel electrophoresis) and loaded with 0.75 µg of protein per slot in non-denaturing loading buffer. The standard for *pro*MMP-2 and *pro*MMP-9 activity was medium from U937 promyelocyte cells (ATCC, CRL-1593.2; Manassas, VA, USA). The samples were diluted with buffer (0.5 M Tris, pH 6.8, 10% glycerol, and 0.1% bromophenol blue). Electrophoresis was carried out at a constant voltage of 25 mA at 4 °C for 90 min. Subsequently, SDS was removed by incubating the sample twice with 2.5% Triton X-100 at room temperature for 15 min under constant agitation. The gels were incubated overnight at 37 °C in activation buffer: 50 mM Tris (pH 7.4), 0.1 M CaCl_2_, 0.15 M NaCl, and 0.2 mg/mL NaN_3_. Afterwards, they were stained for 1 h with 1.0% Coomassie brilliant blue R-250 (Sigma Aldrich, St. Louis, MO, USA) in methanol/acetic acid/glycerol/water (10:10:10:70) at room temperature, and then placed in a methanol/acetic acid/water solution (10:10:80) until intense bands were seen. The activity of the lysis bands (*pro*MMP-2 and *pro*MMP-9) was visualized by densitometry with the EpiChemi Darkroom gel documentation system (UVP; CA, USA). The optical density was quantified using NIH ImageJ software and expressed in relative densitometric units.

### 2.6. Statistical Analysis

All data were analyzed using a Shapiro–Wilk test to assess the normal distribution. The mares, pregnancies, foal features, and MMP activity were analyzed by one-way ANOVA with multiple comparison, followed by Tukey’s test between the HM-HF group and all the other groups (HM-HF vs. USP-HF, HM-HF vs. USP-HF, HM-HF vs. USP-LSeF, and HM-HF vs. USP-DF). *e*IL-1β concentrations were compared by a non-parametric Mann–Whitney U test. All analyses were conducted using GraphPad Prism version 8.0 (GraphPad Software, San Diego, CA, USA). Statistical significance was considered at *p* < 0.05. All values are expressed as the mean ± standard deviation or the median and interquartile range, depending on the data distribution.

## 3. Results

The distribution of cases according to the conditions of the mare during pregnancy (HM, or USP) and the status of the foals (HF, LSeF, and DF) are shown in Table 1.

The average age of the mares was not different among the groups (*p* = 0.5466). Normal and abnormal pregnancies, and the delivery of healthy and unhealthy foals occurred both in young and old mares. The duration of pregnancy was similar among the groups, except for pregnancies that culminated in a dead foal, which were significantly shorter (321.7 ± 11.9, *p* = 0.0235) compared to healthy pregnancies resulting in a healthy foal (344.8 ± 5.3). The shortest pregnancy, which culminated in spontaneous abortion on day 308 of gestation, occurred in a mare with USP-DF (*p* = 0.0235). There were no statistical differences among the groups in the average weight of the foals (*p* = 0.0699; Table 1), even considering the low weight of the aborted foal (32 kg), which reduced the average in the USP group. No other foal in any group weighed less than 42 kg (*p* = 0.2854; Table 1).

### 3.1. Concentration of eIL-1β

**Figure 2:**
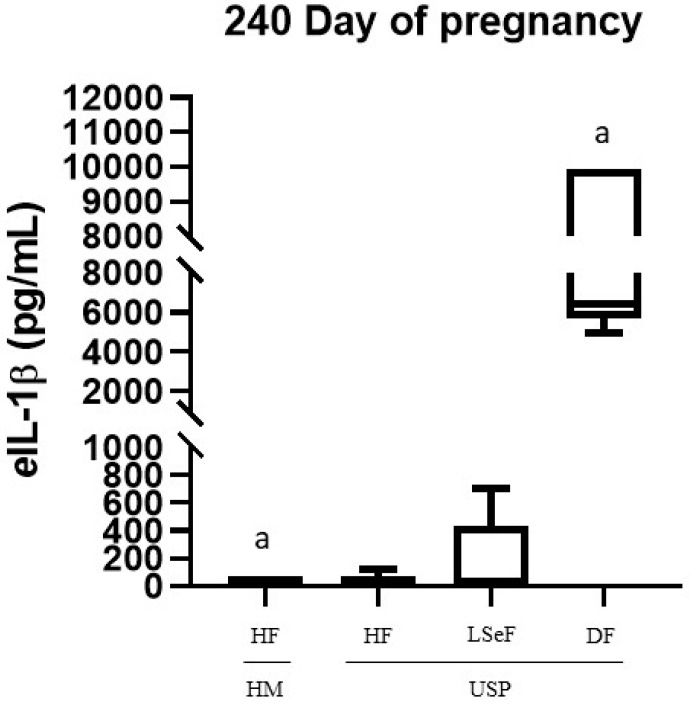
Concentration of *e*IL-1β in the serum of mares at 240 days of pregnancy. Healthy mares (HM; *n* = 6), and mares with ultrasonographic signs of placentitis (USP; *n* = 10). Healthy foals (HF), live septic foals (LSeF), and dead foals (DF). The data are presented as boxes representing the median (central line) with the interquartile range (25th and 75th percentiles), and the whiskers represent the extreme points. Each assay was performed in duplicate. ^a^ *p* < 0.00001.

**Figure 3:**
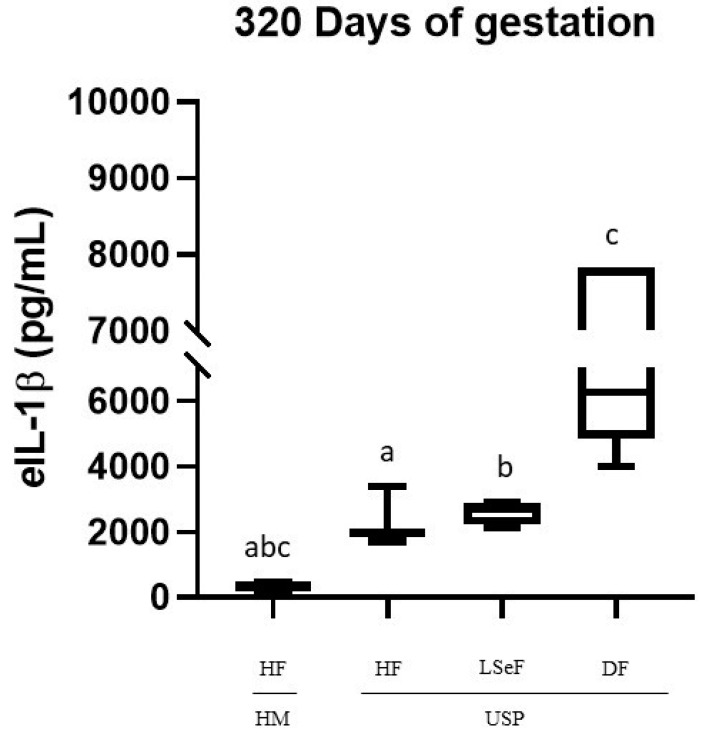
Concentrations of *e*IL-1β in the serum of mares at 320 days of pregnancy. Healthy mares (HM; *n* = 6), and mares with ultrasonographic signs of placentitis (USP; *n* = 10). Healthy foals (HF), live septic foals (LSeF), and dead foals (DF). The data are presented as boxes representing the median (central line) with the interquartile range (25th and 75th percentiles), and the whiskers represent the extreme points. Each assay was performed in duplicate. ^a^ *p* = 0.0002, ^b^ *p* = 0.0006, ^c^ *p* < 0.0001.

The serum concentrations of *e*IL-1β on days 240 and 320 of gestation for each group of mares are shown in Figure 2 and Figure 3, respectively. On day 240 of gestation, the concentration of *e*IL-1β was 20.17 ± 3.8 ng/mL in the group of HM-HF and 2.7-fold higher in the USP-HF group; no statistical significance (ns) was found (HM-HF vs. USP-HF, *p* > 0.9999; ns. Figure 2). Comparing the groups HM-HF and USP-LSeF, the second had a 8.4-fold increase in *e*IL-1β concentrations (HM vs. USP-LSeF, *p* = 0.9958), and when compared to those that delivered a dead foal (USP-DF), it showed a 373.4-fold greater level of *e*IL-1 β (HM vs. USP-DF, *p* < 0.0001; Figure 2).

When comparing USP mares according to the foal data (HF, LSeF, and DF), we found statistically significant differences between USP-HF and USP-DF (*p* < 0.0001). In contrast, we did not find significant differences between USP-LSeF and USP-HF (*p* = 0.9981; Figure 2).

On day 320 of pregnancy, the concentration of *e*IL-1β was significantly different between the groups of HM-HF and USP-HF (*p* = 0.0002), and between mares in the USP group that delivered a live septic foal (HM vs. USP-LSeF, *p* = 0.0006) and those who delivered a dead foal (HM vs. USP-DF, *p* < 0.0001). All showed significantly high levels of *e*IL-1β (Figure 3).

When comparing the USP mares according to the foal data (HF, LSeF, and DF), we found statistically significant differences between USP-LSeF and USP-DF (*p* < 0.0001; Figure 2). However, no statistically significant difference was found between USP-HF and USP-LSeF (*p* = 0.7795).

According to these results, the pathogenic process of spontaneous placentitis in mares appears to activate an inflammatory response mediated by *e*IL-1β, as has been demonstrated in the experimental models of induced placentitis [29]. In those models, extracellular MMPs are activated in the next phase of the inflammatory response [68,69], which is why the lytic activity profile of *pro*MMP-2 and *pro*MMP-9 was determined in the serum of mares in the next step.

### 3.2. proMMP-2 Activity at 240 and 320 Days of Gestation

#### 3.2.1. *pro*MMP-2 Activity at 240 Days of Gestation

**Figure 4:**
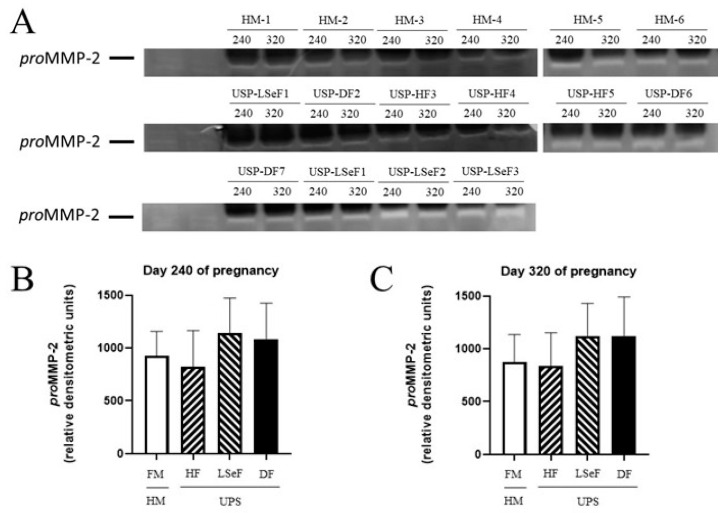
Activity of *pro*MMP-2 in the serum of mares at 240 and 320 days of pregnancy. The activity band of the electrophoretic mobility marker U937 (lanes 1 and 2) resulting from gel zymography is portrayed for the three groups of mares: healthy mares (HM-HF, lanes 1–6), and mares with ultrasonographic signs of placentitis (USP, lanes 1–10). The product of gestation indicates a healthy foal (HF), live septic foal (LSeF), or dead foal (DF) (**A**). The optical density of each lysis band was determined at 240 (**B**) and 320 days of gestation (**C**). Data are expressed as the mean ± standard deviation in relative densitometric units.

On day 240 of pregnancy, the level of *pro*MMP-2 was 924.4 ± 233.2 (relative densitometric units) in the HM-HF group, and 1.1-fold lower in mares in the USP-HF group, (MH-HF vs. USP-HF, *p* = 0.9587; Figure 4B). Comparing the HM-HF to the USP group that delivered a live septic foal showed a 1.2-fold increment (HM-HF vs. USP-LSeF, *p* = 0.6713), while comparing the HM-HF group to mares in the USP group that delivered a dead foal showed a 1.1-fold increment (HM-HF vs. USP-DF, *p* = 0.8681; Figure 4B).

#### 3.2.2. *pro*MMP-2 Activity at 320 Days of Gestation

On day 320 of pregnancy, the level of *pro*MMP-2 was 876.7 ± 257.4 (relative densitometric units) in HM-HF group and 1.04-fold lower in the USP group that delivered a healthy foal (HM vs. USP-HF, *p* = 0.9979, *ns*; Figure 4C). When the HM-HF group was compared to mares of the USP group, an increase of 1.2-fold was found (HM vs. USP-LSeF, *p* = 0.5961). When the HM-HF group was compared to mares in the USP group that delivered a dead foal, 1.27-fold increase was found (HM-HF vs. USP-DF, *p* = 0.6690; Figure 4C).

#### 3.2.3. *pro*MMP-9 Activity at 240 Days of Gestation

**Figure 5:**
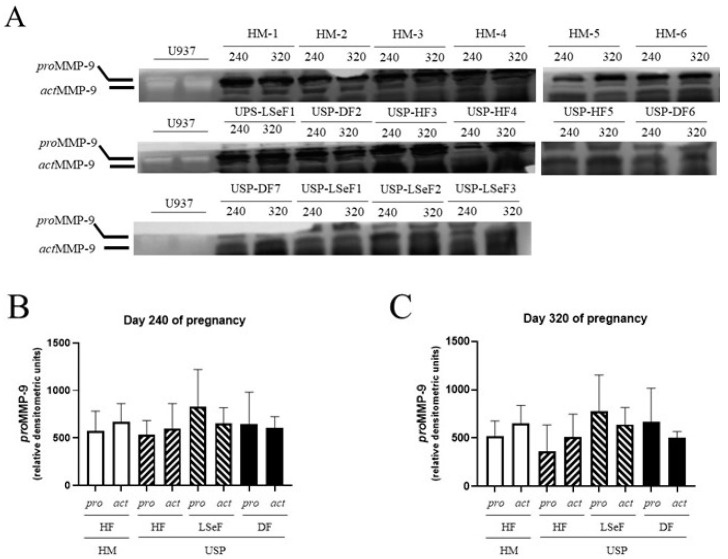
Activity of *pro*MMP-9 and *act*MMP-9 in the serum of mares at 240 and 320 days of pregnancy. The activity band of the electrophoretic mobility marker U937 (lanes 1 and 2) resulting from gel zymography is shown for the three groups of mares: healthy mares (HM-Hf, lanes 1–6), and mares with ultrasonographic signs of placentitis (USP, lanes 1–10). The product of gestation is indicated as a healthy foal (HF), live septic foal (LSeF), or dead foal (DF) (**A**). The optical density of each lysis band was determined at 240 (**B**) and 320 days of gestation (**C**). Data are expressed as the mean ± standard deviation in relative densitometric units (The original pictures can be found in Figure S1).

On day 240 of pregnancy, the level of *pro*MMP-9 activity was 573.2 ± 209.6 (relative D.O units) in the HM-HF group and 1.06-fold lower in mares with the USP group that delivered a healthy foal (HF vs. USP-HF, *p* = 0.9999; Figure 5B). Comparing the HM-HF to the USP group that delivered a live septic foal, an increment of 1.4-fold was found (HM-HF vs. USP-LSeF, *p* = 0.7143), and when the HM-HF group was compared to the USP group that delivered a dead foal, a 1.12-fold increase was found, (HM-HF vs. USP-DF, *p* = 0.9999).

#### 3.2.4. *act*MMP-9 Activity at 240 Days of Gestation

On day 240 of pregnancy, the activity of the active isoform (*act*) of MMP-9 was 671.9 ± 191.1 (relative D.O units) in the HM-HF group (Figure 5B) and 1.12-fold lower in the USP group that delivered a healthy foal (HM-HF vs. USP-HF, *p* = 0.9819; Figure 5B). Mares with USP delivering a live septic foal and those delivering a dead foal showed 1.19-fold (HM-HF vs. USP-LSeF, *p* = 0.9838) and 1.10-fold (HM-HF vs. USP-DF, *p* = 0.9883) higher activity of actMMP-9 (Figure 5B).

#### 3.2.5. *pro*MMP-9 Activity at 320 Days of Gestation

On day 320 of pregnancy, the activity of *pro*MMP-9 was 517.2 ± 160.3 (relative D.O units) in the HM-HF group (Figure 5B) and 1.41-fold lower in mares in the USP group that delivered a healthy foal (HM-HF vs. USP-HF, *p* = 0.9460; Figure 5C). Comparing the group of HM-HF to the USP group that delivered a live septic foal and those that delivered a dead foal showed 1.5-fold (HM-HF vs. USP-LSeF, *p* = 0.9186) and 1.28-fold (HM-HF vs. USP-DF, *p* = 0.9486) higher *pro*MMP-9 activity (Figure 5C).

#### 3.2.6. *act*MMP-9 Activity at 320 Days of Gestation

On day 320 of pregnancy, the activity of *act*MMP-9 was 655.5 ± 182.2 (relative D.O units) in the HM-HF group (Figure 5C), and 1.28-fold lower in the USP group that delivered a healthy foal (HM-HF vs. USP-HF, *p* = 0.7800; Figure 5C). Comparing HM-HF mares to mares in the USP group that delivered a live septic foal and those that delivered a dead foal exhibited 1.29-fold (HM-HF vs. USP-LSeF, *p* = 0.9341) and 1.29-fold (HM-HF vs. USP-DF, *p* = 0.7649) decreased *act*MMP-9 activity (Figure 5C).

## 4. Discussion

Equine placentitis is one of the main causes of abortion [70,71], mare mortality, premature activation of labor, and foal mortality [2,63]. The models of equine placentitis involving the inoculation of pathogenic bacteria (*Streptococcus equi* subspecies *zooepidemicus*, *Escherichia coli*, and/or beta-hemolytic *Streptococcus dysgalactiae*) [4,29,72,73,74] have demonstrated that infection-induced inflammation activates a complex signaling network, which leads to the production of IL-1β, IL-6, TNFα [29,30], IL-8 [2,75], and prostaglandins E2 and F2 [31]. Additionally, the induced inflammation increases the level of degradative metalloproteinases (MMPs) in the amniotic fluid [76,77] and the expression of genes involved in placental regulation (PLAC8, PAPPA, and LGALS1) [78,79].

Several studies have been carried out on the role of inflammatory molecules in preterm and term labor in humans [80,81], but there is little information on the activation of these inflammatory biomarkers during normal equine gestation or as result of spontaneous placentitis. Hence, the aim of the current contribution was to evaluate inflammatory biomarkers during gestation in mares with and without placentitis. Three biomarkers were measured in mare serum at 240 and 320 days of gestation: the proinflammatory *e*IL-1β and the activity of collagenolytic *pro*MMP-2 and *pro*MMP-9. In healthy mares, the concentration of *e*IL-1β underwent a significant 16.5-fold increase from day 240 to day 320 of gestation. In mares with USP, the concentration of *e*IL-1β showed a similar change during the pregnancies, leading to the delivery of a healthy foal.

### 4.1. The Inflammatory Response and the Activation of Labor

With experimental models of equine and human placentitis, it has been established that the inflammatory response is mediated by *e*IL-1β or IL-1 β [29,68]. The current study reveals that this is similar in spontaneous equine placentitis. *e*IL-1β modulates the onset of labor in mares by inducing placental inflammation through the expression of TNFα [80,82] and chemotactic cytokines (IL-6, IL-8) [81]. These molecules amplify the inflammatory response by recruiting professional antigen-presenting cells of the immune system [83]. The inflammatory response stimulates the synthesis of prostaglandin (PGE) and oxytocin, which, together, initiate uterine contractions [84].

**Figure 6:**
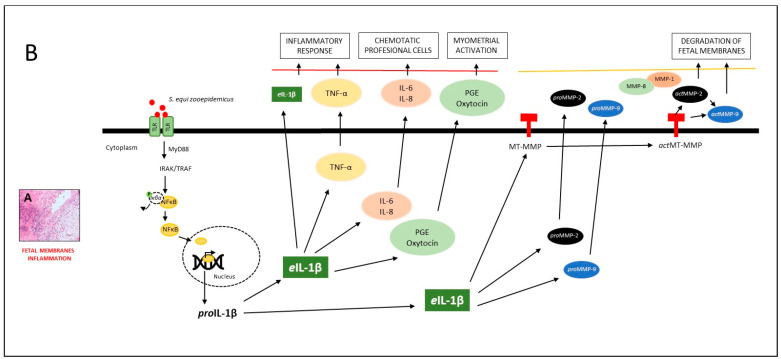
Model of the effect of *e*IL-1β and *pro*MMP-9 in mares with placentitis. (**A**) Histological section of equine placental inflammation is illustrated. (**B**) According to the model, TOLL-like receptors (TLR-2 and TLR-7) [23,24] recognize different pathogen-associated molecular patterns (PAMPs), such as flagellin, lipoproteins, LPS, DNA, and RNA [22]. They then induce the phosphorylation of IRAK/TRAF, which [24] activates the nuclear factor kappa-light-chain-enhancer of activated B cells (NFĸB), facilitating the transcription of *e*IL-1β [26]. The latter has been associated with the inflammatory response (TNFα) [27,28] and chemotactic cytokines (IL-6, IL-8). These molecules amplify the inflammatory response by recruiting professional antigen-presenting cells of the immune system [46,47,50]. The inflammatory response stimulates the synthesis of prostaglandin (PGE) and oxytocin, which, together, initiate uterine contractions. In the next phase of the inflammatory response, *e*IL-1β triggers the activation of *pro*MMP-2 and *pro*MMP-9 [40], leading to the degradation of different structures of the fetal membranes. This results in the weakening of the tensile strength of these membranes, and therefore, the onset of premature labor [49,52].

In the next phase of the inflammatory response, as evidenced in several models of infection, extracellular MMPs are activated [68,69]. These proteins favor a key step that involves uterine contractions and the rupture of the chorionic and amniotic membranes (Figure 6).

Thus, the activity profiles of *pro*MMP-2 and *pro*MMP-9 were determined in this study, as reported in other models of infection. No association was found between spontaneous equine placentitis and the serum concentrations of these metalloproteinases (Figure 5).

The alterations in *pro*MMP-2 and *pro*MMP-9 observed in experimental models of infection in human placental tissues were measured in the tissues themselves [68]. In contrast, alterations identified during labor after normal or high-risk pregnancies in mares were examined in amniotic fluid [77]. Consequently, the lack of a significant difference between groups in the concentrations of *pro*MMP-2 and *pro*MMP-9 in maternal serum in this study does not rule out possible alterations at the level of tissues or fetal fluids.

In human pregnancies, IL-1β is one of the proinflammatory molecules upregulated to prepare for term labor [85]. Our data suggest the existence of a similar process in mares, as evidenced by the robust 16.5-fold increase in the concentration of *e*IL-1β between days 240 and 320 in normal pregnancies (healthy mares). The expression of this cytokine was exacerbated by the degree of inflammation in the mares with placentitis that later did not give birth to a healthy foal. This is evidenced by the much higher concentrations of *e*IL-1β (at 240 and 320 days of pregnancy) in these animals compared to the levels found in healthy mares and in those with USB that delivered healthy foals (Figure 2 and Figure 3).

Interestingly, the concentration of *e*IL-1β was not significantly different between healthy mares (that delivered a healthy foal) and mares with USP that also delivered healthy foals. This suggests that ultrasonography may lead to false positives or may be able to detect slight inflammation that does not affect the fetus. On the other hand, there was a significant difference between the level of *e*IL-1β during pregnancy when comparing healthy mares to those with USP that delivered a live septic neonatal foal, indicating the presence of clinically significant inflammation in the latter despite the lack of macroscopic damage to the placenta collected at the time of delivery. This suggests that the concentrations of *e*IL-1β could be used to determine whether or not a mare with USP is at risk of delivering a septic or dead foal.

### 4.2. The Inflammatory Response to the Infectious Process

A high secretion of *e*IL-1β was detected after the experimental induction of equine placentitis and in cases of mares with retained placenta.

Both were associated with elevated expressions of IL-6, IL-8, and TNFα at the fetal–maternal interface [2,29,30,75,82]. The activation of this inflammatory pathway affects the immune tolerance of the fetus, triggering premature birth with a low probability of survival [80].

LeBlanc et al. (2012) *infected* pregnant mares with 1 × 10^8^ CFU/mL of *Streptococcus equi* subspecies *zooepidemicus*, finding increases in the concentrations of *e*IL-1β, IL-6, TNFα, and PGE2, and PGF2α in the allantoic fluid. These changes were associated with the premature activation of labor, leading to abortions in 87.5% of the mares by day 309 of gestation [29]. Coinciding with the aforementioned study, the upregulation of *e*IL-1β found in this study in most mares with USP at days 240 (Figure 2) and 320 of gestation (Figure 3) was associated with foal infection and mortality (Table 1).

Recently, Fedorka et al. (2019) intracervically inoculated *S. zooepidemicus* to pregnant mares and found significant increases in the concentrations of *e*IL-1β, *e*IL-6, and *e*IL-10 in the amniotic fluid, but not in the serum of mares or neonatal foals [2]. In the current contribution, we found an elevated concentration of *e*IL-1β in the serum of mares with USP that delivered a live septic or dead foal, but not in those that delivered a healthy foal (Figure 2 and Figure 3).

Our results suggest that the quantification of *e*IL-1β and *pro*MMPs in the serum of pregnant mares offers a non-invasive alternative that will help veterinarians to confirm the diagnosis of placentitis, reducing possible complications for both the pregnant mare and the foal. The determination of these biomarkers is fast, low-cost, and does not require specialized personnel or expensive equipment.

## 5. Conclusions

In this study, we demonstrated that *e*IL-1β levels in the serum of mares with ultrasonographic signs of placentitis can be used as biomarkers of disease severity and its probable impact on the heath and viability of the foal.

## Figures and Tables

**Table 1:** Mares, pregnancies, and foal features.

		HEALTHY MARES (*n* = 6)	USP (*n* = 10)		
Variable		HM-HF (*n* = 6)	USP-HF (*n* = 3)	USP-LSeF (*n* = 4)	USP-DF (*n* = 3)
*Mares*					
Age, years (Rank)		12.5 ± 4.8 (9–22)	9.3 ± 4.2 (6–14)	10.5 ± 3.7 (6–14)	14.3 ± 5.5 (8–10)
CTUP, mm	240 days	3.98 ± 0.55	5.0 ± 1.3	5.7 ± 0.98 *	5.8 ± 1.0 *
	320 days	8.56 ± 1.93	9.0 ± 0.5	10.8 ± 0.8	12.1 ± 0.59 *
External signs of placentitis		Normal	Normal	Normal	Premature udder and vaginal discharge
Delivery, days (Rank)		344.8 ± 5.3 (338–350)	337. ± 11.9 (329–351)	334.3 ± 11.5 (325–351)	321.7 ± 11.9 ^a^ (308–330)
Placental lesions		Normal	Ascending lesions	Ascending lesions	Ascending lesions, with necrosis
*Foal*					
Sex	Male, *n* (%)	4 (66.7)	2 (66.7)	0	2 (66.7)
	Female, *n* (%)	2 (33.3)	1 (33.3)	4 (100)	1 (33.3)
Weight, kg (rank)		51.3 ± 5.2 (44–57)	44.3 ± 7.6 (32–50)	41.3 ± 7.3 (31–49)	44.0 ± 1.0 (43–45)

Abbreviations: HM, healthy mare; USP, ultrasonographic signs of placentitis; HF, healthy foal; DF, dead foal; CTUP, combined thickness of the uterus and the placenta. Values are expressed as the mean ± standard deviation. * Represents statistical differences (*p* < 0.05) compared to HM-HF.

## Data Availability

All the relevant information from the study is described in the manuscript.

## Supplementary Materials

The following supporting information can be downloaded at: https://www.mdpi.com/article/10.3390/vetsci10090532/s1, Figure S1: The original pictures of Figure 5A.
