# Supplementary material for: Equine Placentitis in Mares Induces the Secretion of Pro-Inflammatory Cytokine eIL-1β and the Active Extracellular Matrix Metalloproteinase (MMP)-9
Source: Vet Sci. 2023 Aug 22;10(9):532. doi: 10.3390/vetsci10090532 (PMC10536981; doi:10.3390/vetsci10090532)
Supplement: Supplementary file 1 [file vetsci-10-00532-s001.zip › vetsci-2487156-supplementary.pptx]

## Slide 1
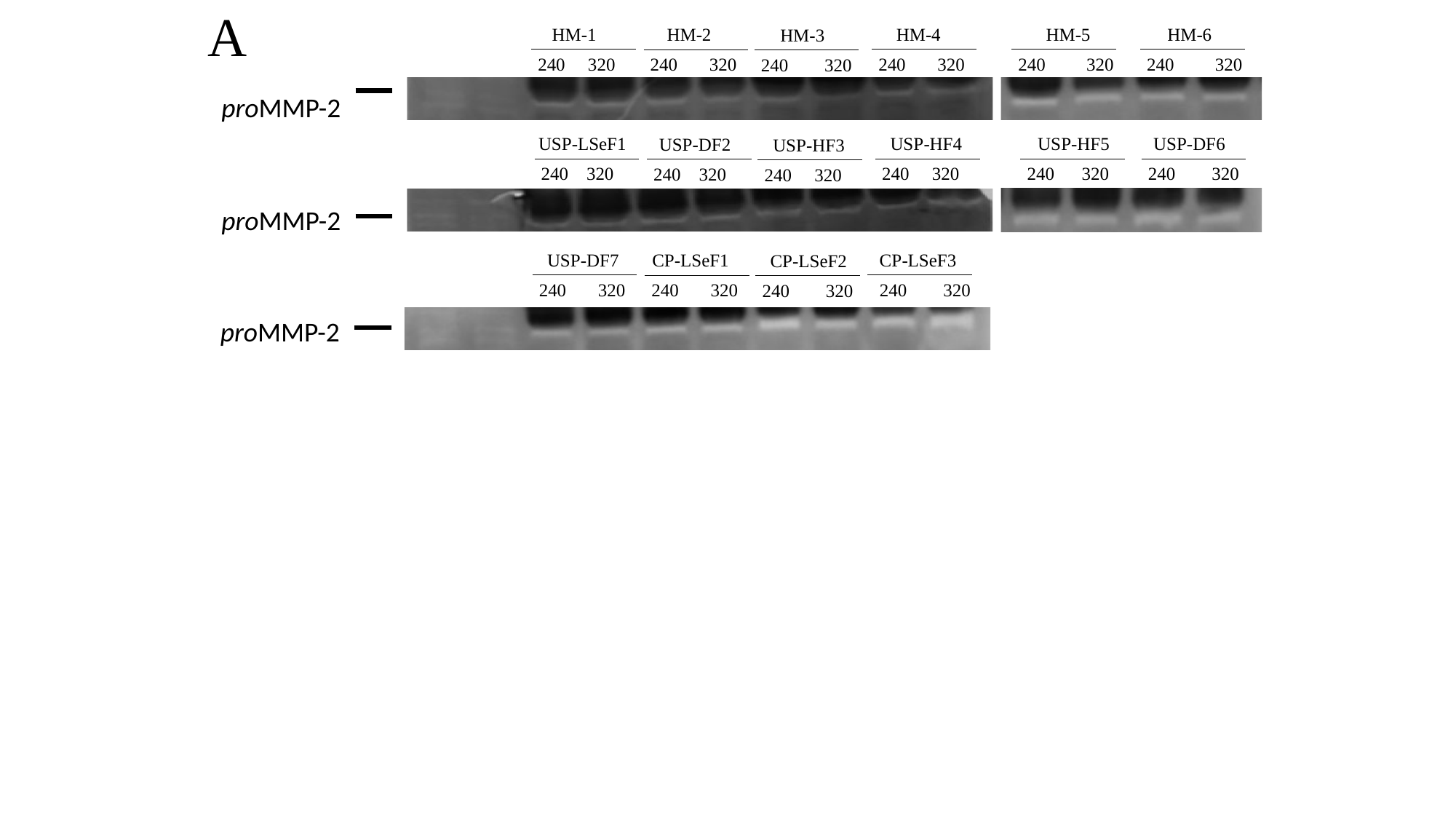

A
HM-1
HM-4
HM-5
HM-6
HM-2
HM-3
 240 320
 240 320
 240 320
 240 320
 240 320
 240 320
proMMP-2
USP-LSeF1
USP-HF4
USP-HF5
USP-DF6
USP-DF2
USP-HF3
 240 320
 240 320
 240 320
 240 320
 240 320
 240 320
proMMP-2
USP-DF7
CP-LSeF3
CP-LSeF1
CP-LSeF2
 240 320
 240 320
 240 320
 240 320
proMMP-2

## Slide 2
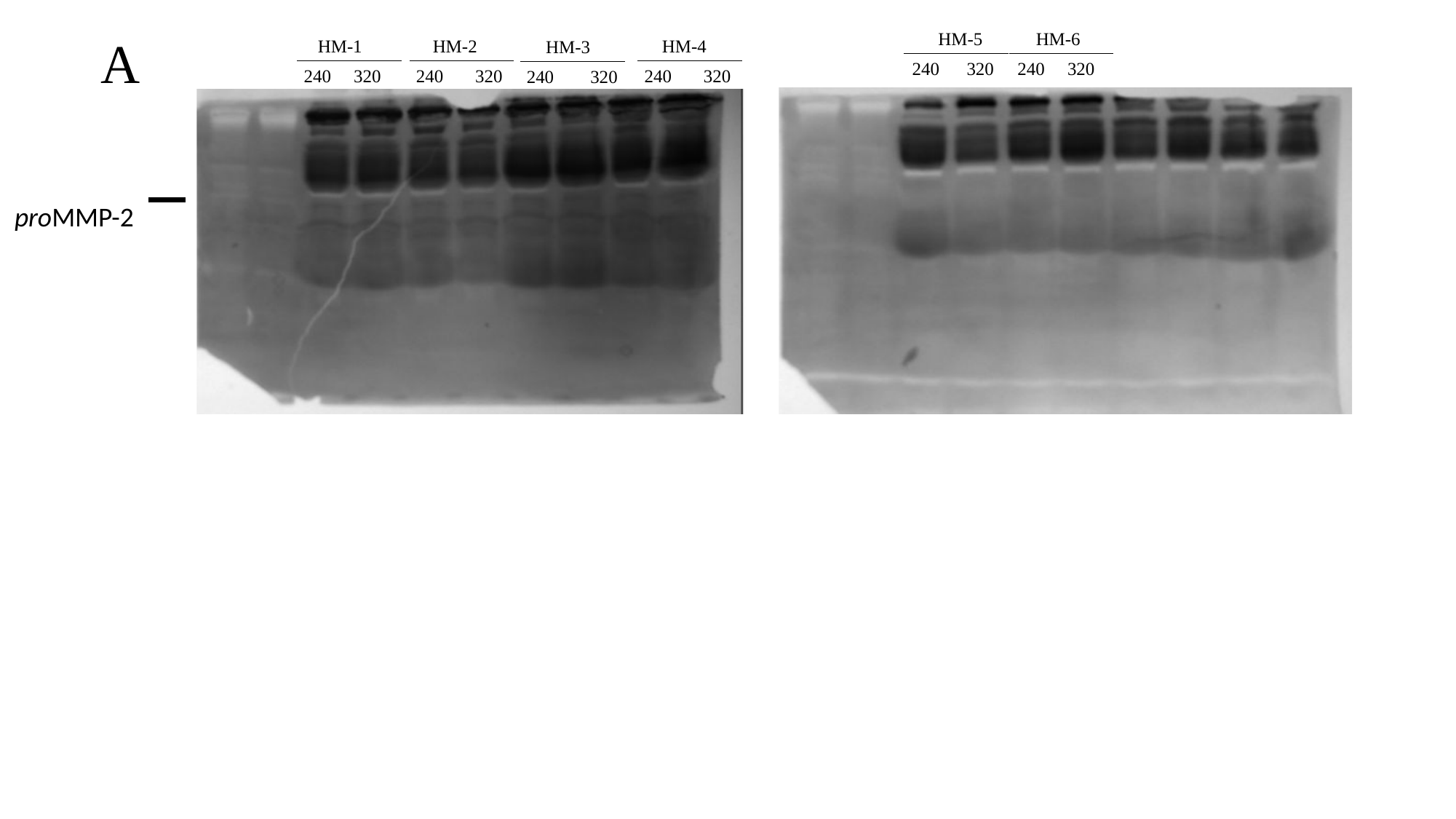

HM-5
HM-6
A
HM-1
HM-4
HM-2
HM-3
 240 320
 240 320
 240 320
 240 320
 240 320
 240 320
proMMP-2

## Slide 3
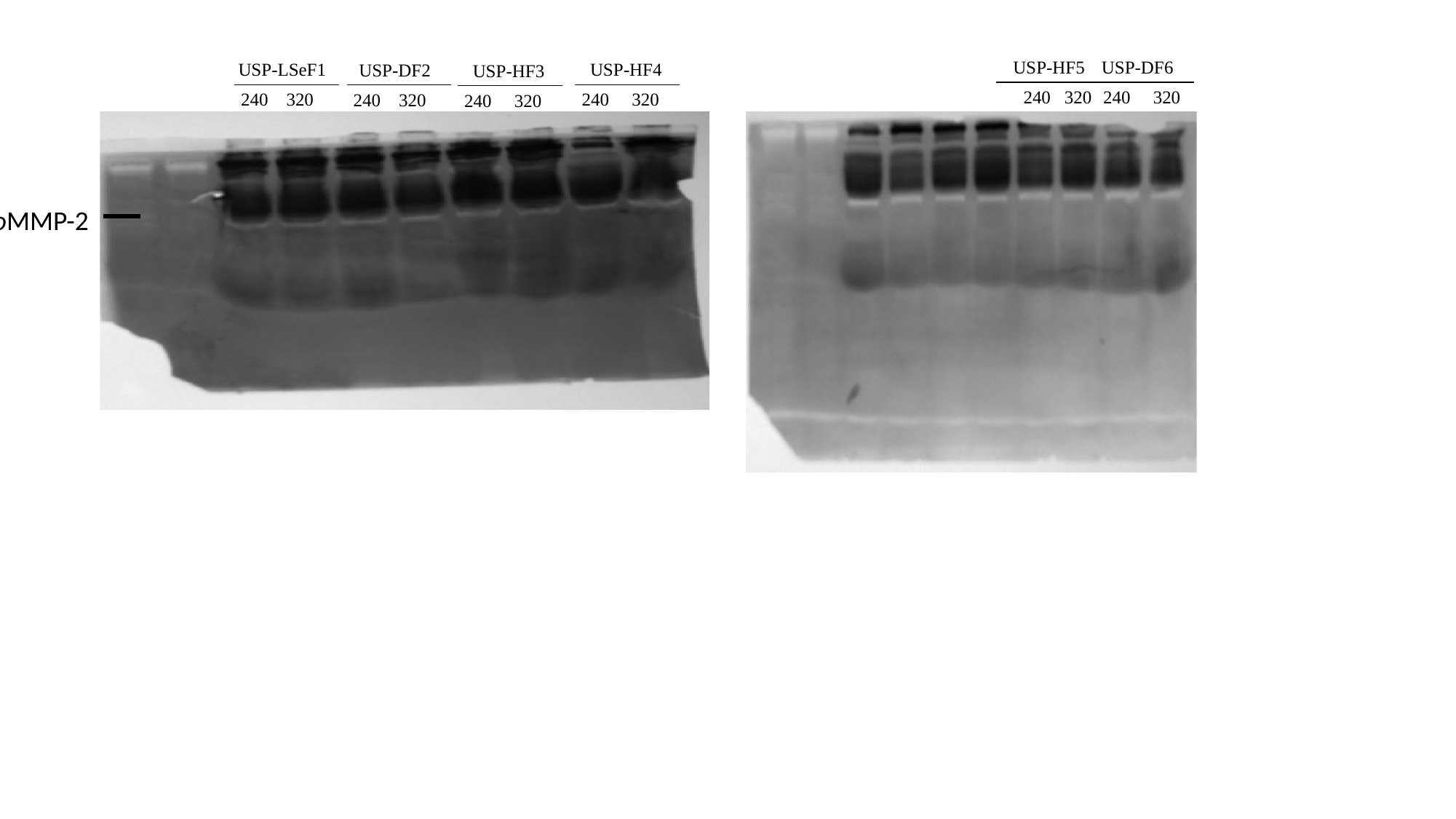

USP-HF5
USP-DF6
USP-LSeF1
USP-HF4
USP-DF2
USP-HF3
 240 320
 240 320
 240 320
 240 320
 240 320
 240 320
proMMP-2

## Slide 4
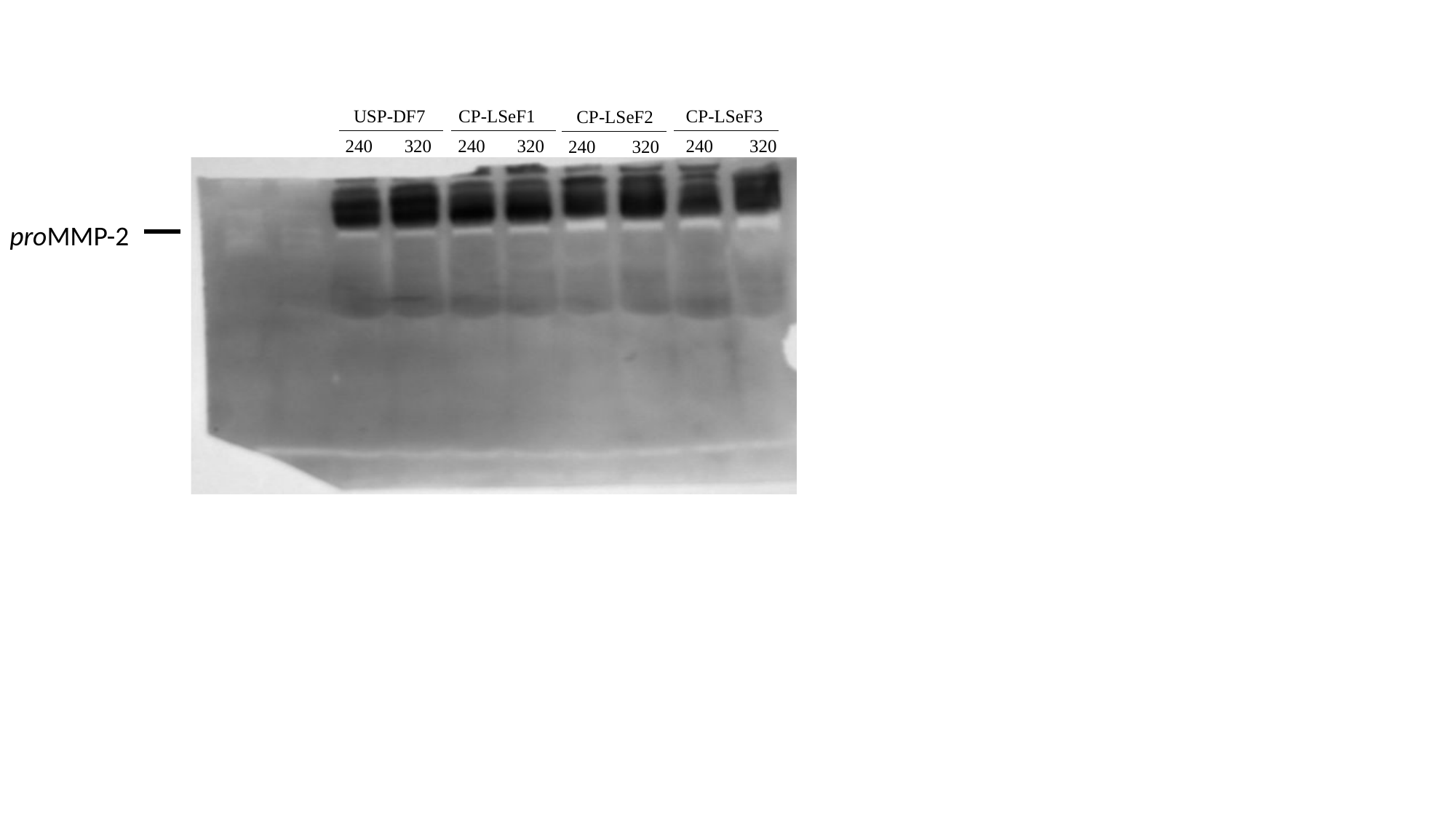

USP-DF7
CP-LSeF3
CP-LSeF1
CP-LSeF2
 240 320
 240 320
 240 320
 240 320
proMMP-2

## Slide 5
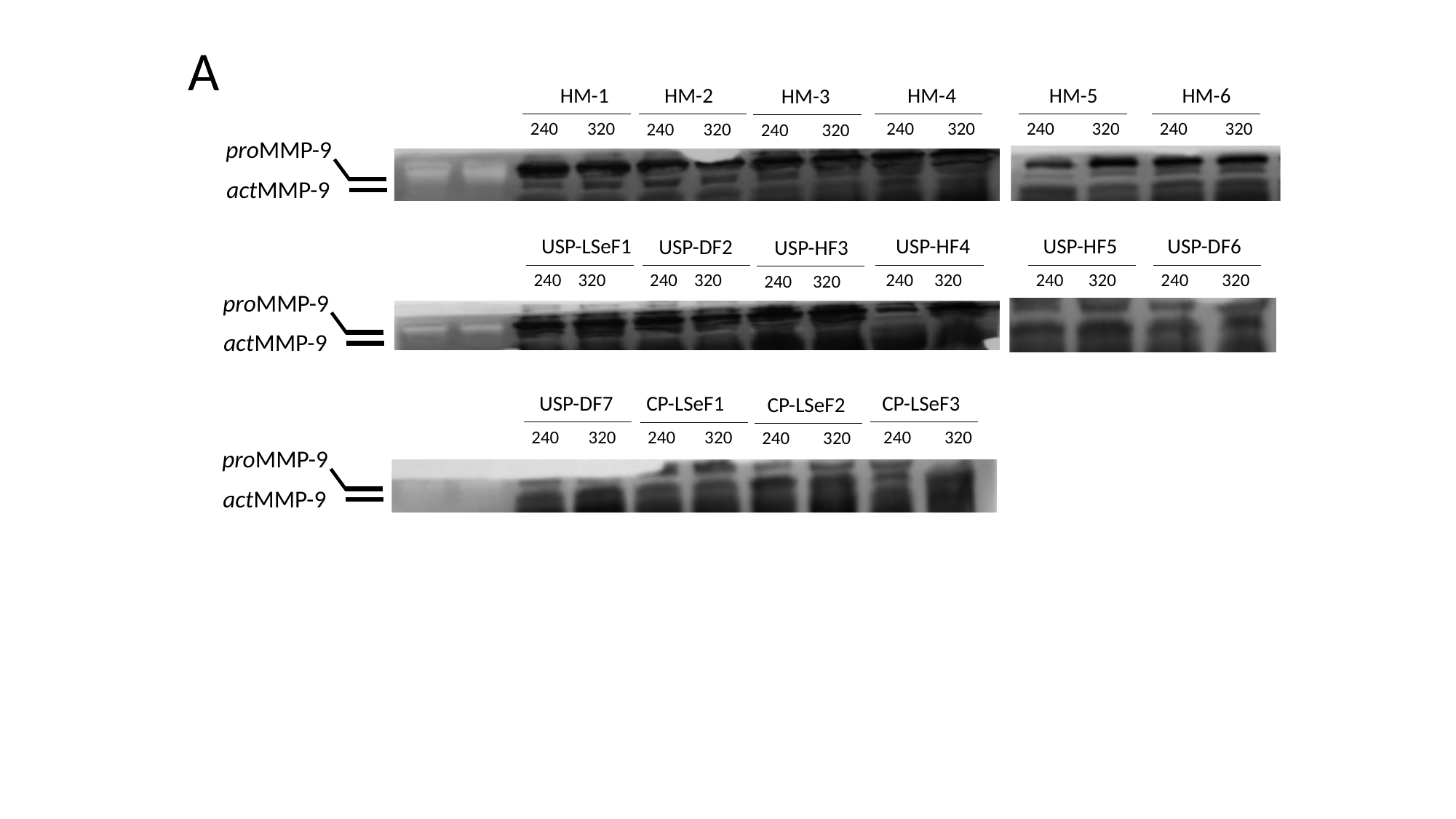

A
HM-1
HM-4
HM-5
HM-6
HM-2
HM-3
 240 320
 240 320
 240 320
 240 320
 240 320
 240 320
proMMP-9
actMMP-9
USP-LSeF1
USP-HF4
USP-HF5
USP-DF6
USP-DF2
USP-HF3
 240 320
 240 320
 240 320
 240 320
 240 320
 240 320
proMMP-9
actMMP-9
USP-DF7
CP-LSeF3
CP-LSeF1
CP-LSeF2
 240 320
 240 320
 240 320
 240 320
proMMP-9
actMMP-9

## Slide 6
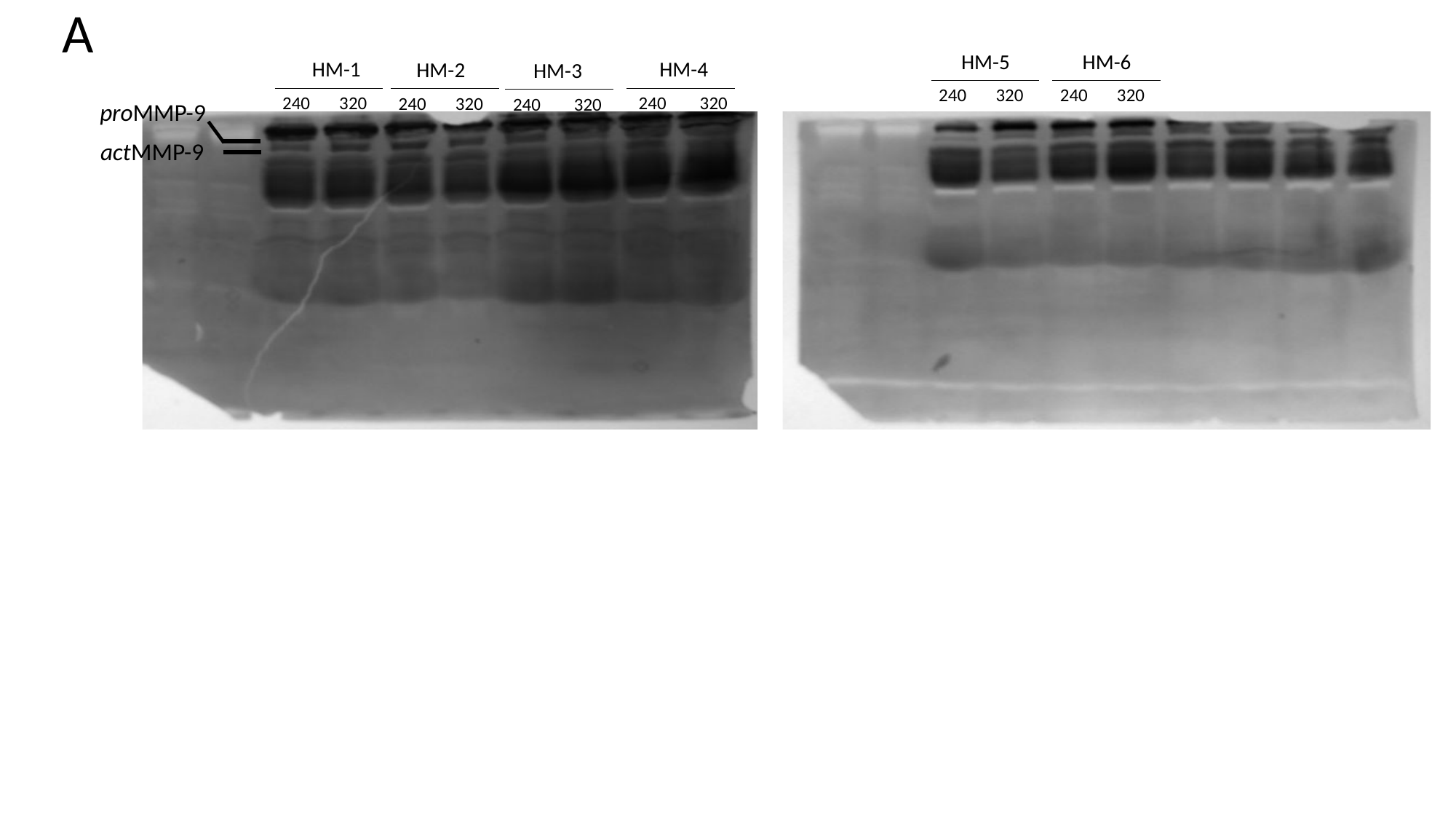

A
HM-5
HM-6
HM-1
HM-4
HM-2
HM-3
 240 320
 240 320
 240 320
 240 320
 240 320
 240 320
proMMP-9
actMMP-9

## Slide 7
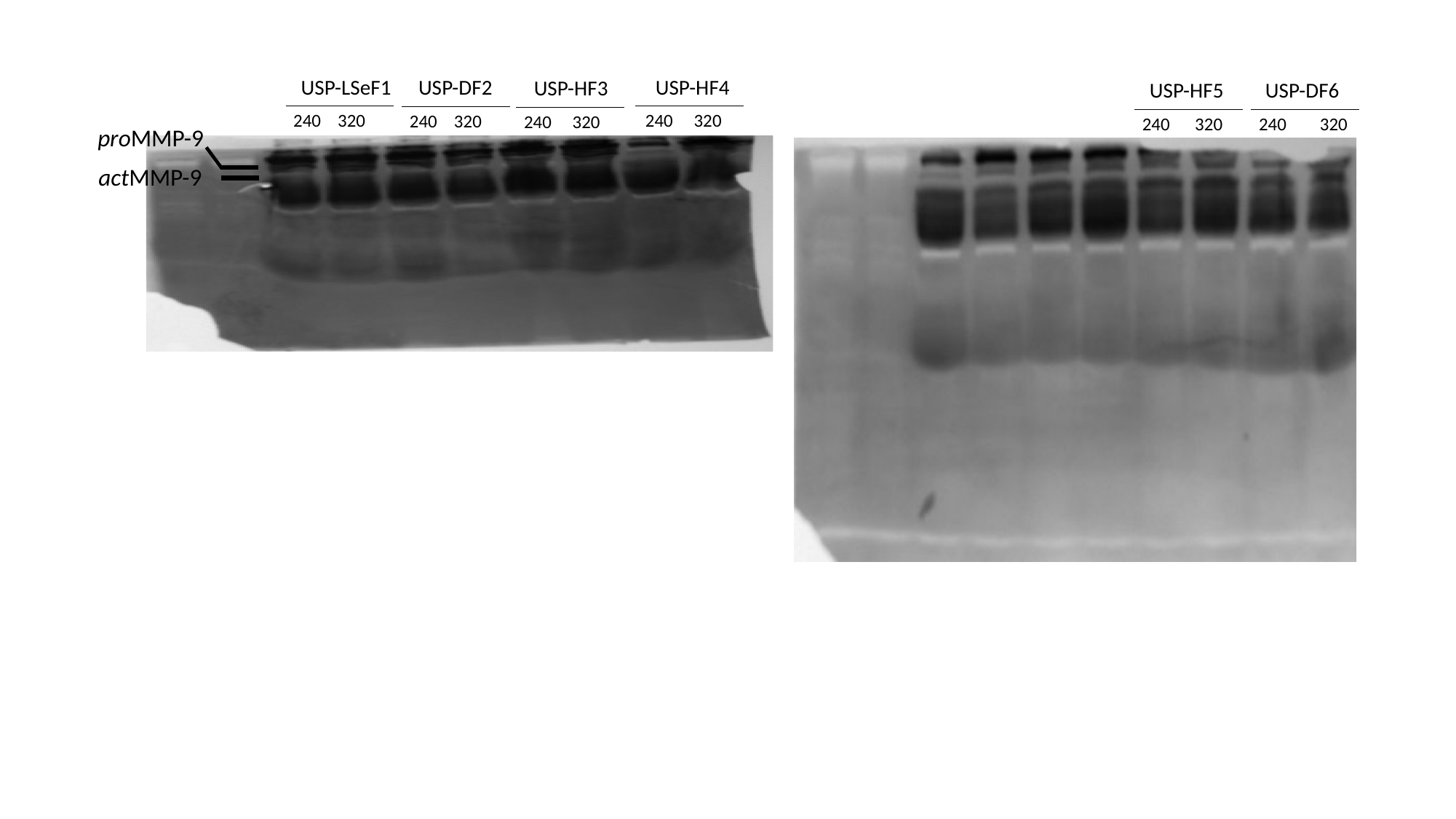

USP-LSeF1
USP-HF4
USP-DF2
USP-HF3
USP-HF5
USP-DF6
 240 320
 240 320
 240 320
 240 320
 240 320
 240 320
proMMP-9
actMMP-9

## Slide 8
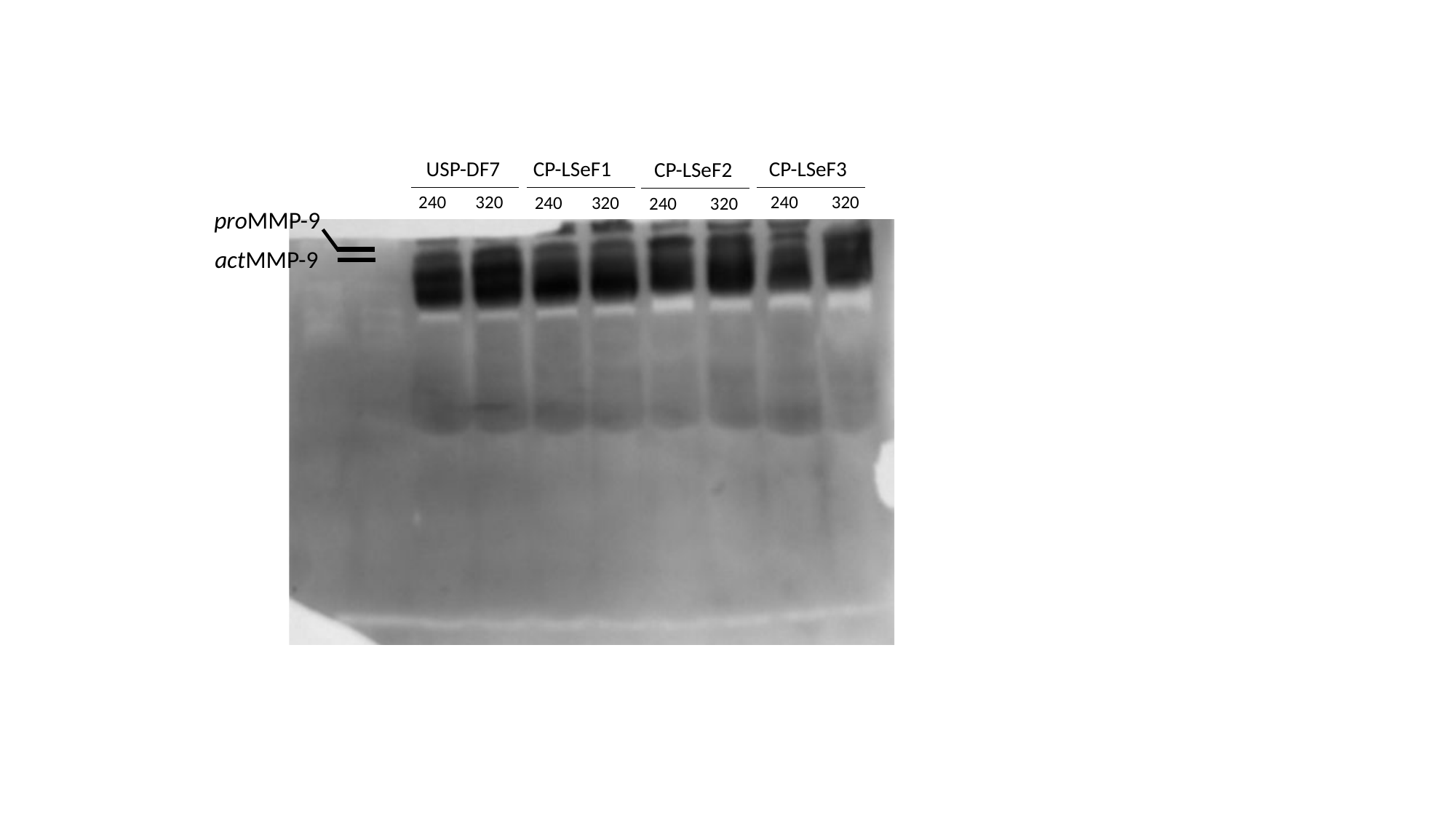

USP-DF7
CP-LSeF3
CP-LSeF1
CP-LSeF2
 240 320
 240 320
 240 320
 240 320
proMMP-9
actMMP-9
